# USP10 in Neurological Disorders: Mechanistic Insights and Emerging Therapeutic Strategies

**DOI:** 10.1111/nyas.70144

**Published:** 2025-11-24

**Authors:** Celemuge, Hongying Sun, Jia Zhang, Yang Yang, Jian Mao, Cheliger

**Affiliations:** ^1^ Baotou Medical College Baotou China; ^2^ The First Affiliated Hospital of Baotou Medical College Baotou China

**Keywords:** deubiquitination, neurological disorders, structure, therapeutic strategies, USP10

## Abstract

Various cellular processes, such as DNA repair and signal transduction, are regulated through ubiquitination and deubiquitination. Dysregulation of ubiquitination cascade enzymes and deubiquitinating enzymes leads to various diseases. Among them, deubiquitinating enzymes have been shown to be closely associated with cancer, cardiovascular disease, and metabolic diseases. Recent studies have found that deubiquitinating enzymes play an important role in controlling neuronal fate, synaptic development, and maintaining normal nervous system function. USP10, a member of the deubiquitinating enzyme family, regulates the progression of various diseases by acting on different substrates and modulating their functions. USP10 has been shown to regulate neurological diseases by mediating pathways such as immune response, oxidative stress, and apoptosis. This review provides a comprehensive overview of the molecular structure of USP10, identifies its substrate‐binding sites, and summarizes its biological functions, particularly in relation to neurological diseases, including Alzheimer's disease, Parkinson's disease, glioblastoma, and ischemic stroke. USP10 promotes pathological progression in Alzheimer's disease and glioblastoma on the one hand, and exerts protective effects in Parkinson's disease and ischemic stroke on the other. Additionally, we summarize recent progress in the development and application of USP10 modulators and potential therapeutic strategies targeting USP10 in neurological disorders.

## Graphical abstract

USP10 is a deubiquitinating enzyme that affects neurological diseases through multiple mechanisms, including the accumulation of toxic proteins, autophagy, and immune responses. In this review, we discuss the structure and characteristics of USP10 and summarize the role of USP10 in neurological disorders. We also further explore recent advances in targeting USP10 with small molecule modulators.

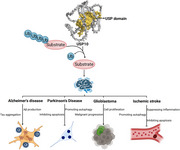

## 1 Introduction

**FIGURE 1:**
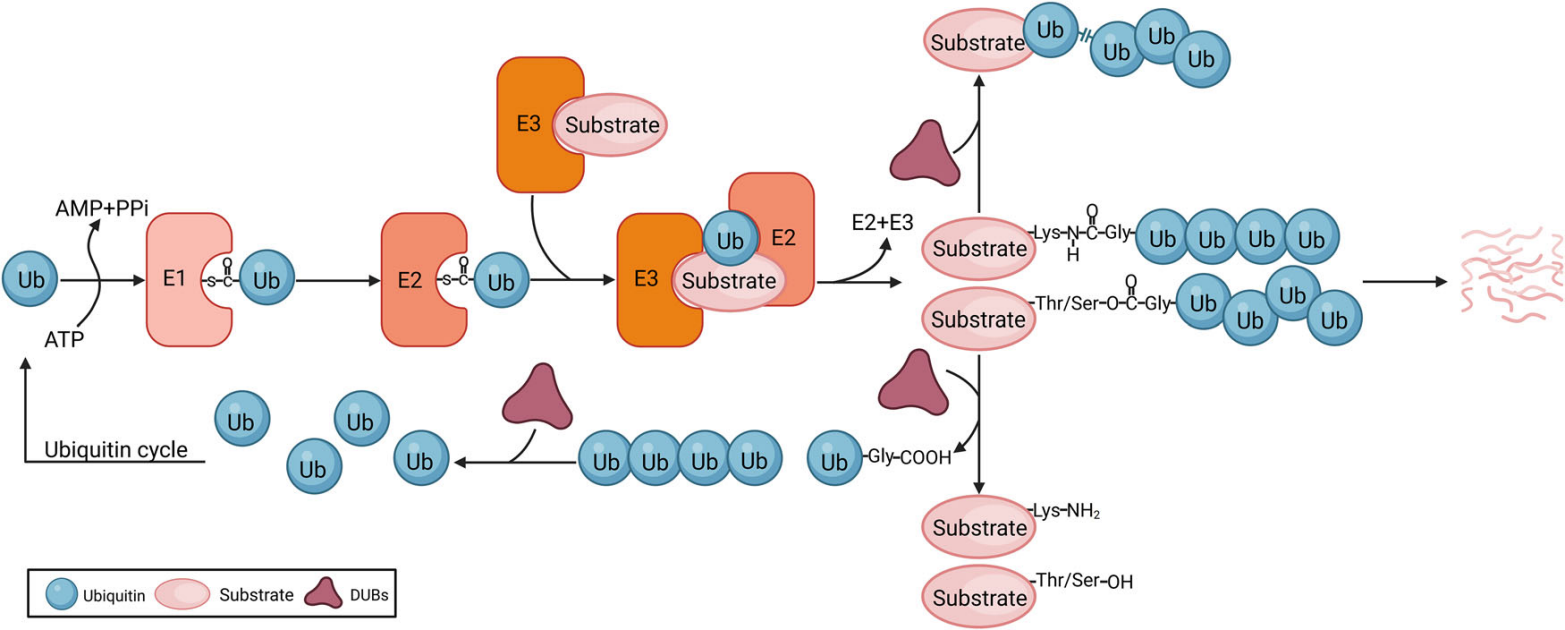
Ubiquitin‐proteasome system. E1 consumes ATP to activate ubiquitin via thioester bond between ubiquitin's C‐terminus and E1's cysteine. The activated ubiquitin molecule is transferred to the cysteine residue of the E2. The E2 facilitates the transfer of ubiquitin to substrate proteins through specific target protein recognition by the E3. Ub is primarily conjugated to substrate proteins via isopeptide bonds between its C‐terminal glycine(Gly) residue and the amino group of lysine(Lys) residue. In rare cases, Ub forms the ester bond with hydroxyl groups of serine or threonine residues on target proteins. DUBs stabilize substrate proteins by cleaving ubiquitin chains attached to them and preventing their recognition and degradation by the proteasome. DUBs can also modulate polyubiquitin chains and regulate the functional outcomes of ubiquitin signaling.

Diverse biological processes, including gene expression, cell metabolism, and immune responses, are regulated through post‐translational modifications, such as phosphorylation, methylation, and ubiquitination [1]. Following ubiquitination, proteins are recognized and degraded by proteasomes. This process is crucial for maintaining cellular protein homeostasis. Additionally, these ubiquitinated proteins mediate protein translocation, DNA repair, and signal transduction [2]. More than 90% of proteins are modified by the ubiquitinating enzymes, and ubiquitination requires the sequential action of three enzymes [3] (Figure 1). In an ATP‐dependent reaction, the ubiquitin‐activating enzyme (E1) activates ubiquitin by forming a high‐energy thioester bond with it. Ubiquitin is subsequently transferred from E1 to the ubiquitin‐conjugating enzyme (E2) through transthiolation. Finally, ubiquitin ligase (E3) recognizes the substrate and attaches ubiquitin to the residue (commonly lysine, threonine, or serine) of the target protein [4]. The substrate can be modified with multiple ubiquitin molecules to form ubiquitin chains or polyubiquitin [5].

**FIGURE 2:**
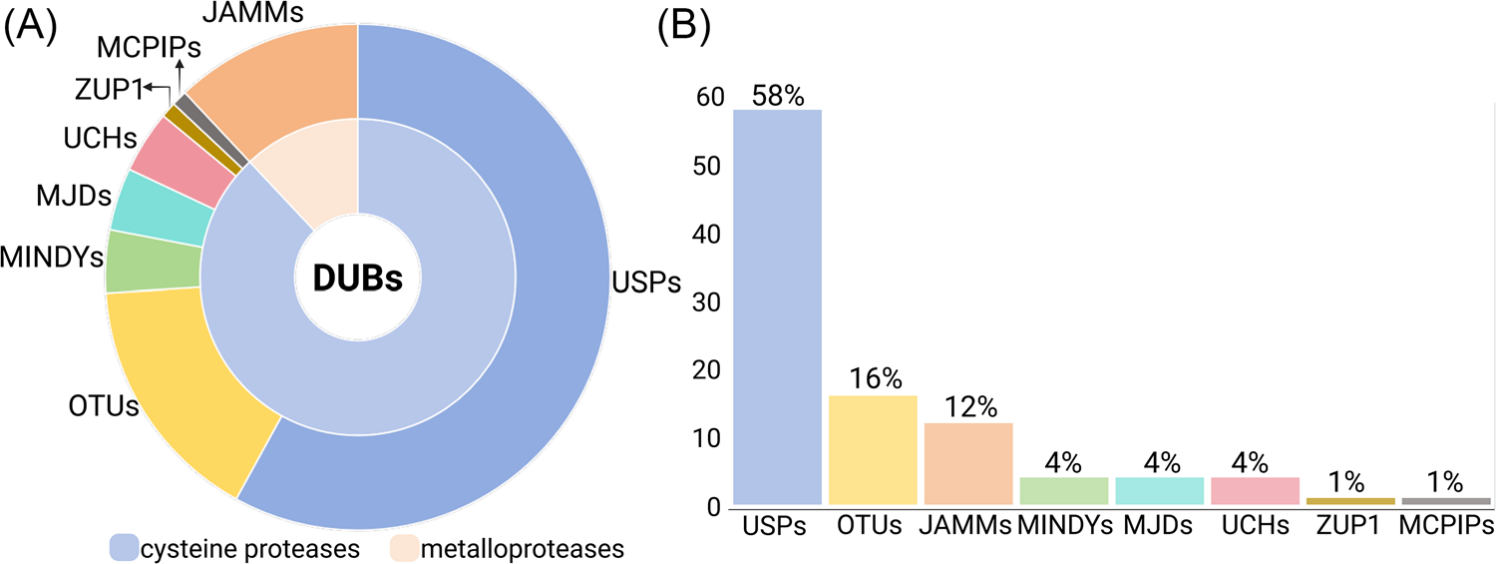
Classification of deubiquitinases.

Ubiquitination is a reversible process. The deubiquitinating enzymes (DUBs) hydrolyze isopeptide and ester bonds between ubiquitin and proteins, as well as between ubiquitin molecules in polyubiquitin chains. This process alters the fate of the substrate proteins [6]. The free ubiquitin molecules produced are reused in subsequent ubiquitination reactions [7]. Deubiquitination is a crucial step in maintaining and regulating cellular processes, including gene transcription, protein hydrolysis, protein activation, cell cycle progression, cell differentiation, and apoptosis [8]. DUBs are a large family of proteases. The human genome encodes approximately 100 DUBs, which are categorized into two types according to their catalytic mechanisms [9] (Figure 2). The first type are cysteine proteases, including ubiquitin‐specific proteases (USPs), ovarian tumor proteases, ubiquitin carboxyl‐terminal hydrolases, Machado−Joseph disease proteases, zinc‐finger‐containing ubiquitin peptidase 1, monocyte chemotactic protein‐induced proteases, and motif‐interacting with ubiquitin‐containing novel DUB family proteases. The second type are metalloproteases, which are represented by the Jab1/Mov34/Mpr1, Pad1 N‐terminal domain proteases [7, 10]. Among them, USPs are the most prominent DUBs, representing the largest subfamily, consisting of over 60 members and serving as key models for studying DUB functions [11].

USP10, short for ubiquitin‐specific peptidase 10, belongs to the USP family. The USP10 gene (*USP10*) is located on human chromosome 16q24.1. The protein encoded by this gene has a conserved structure composed of multiple functional domains, among which the most distinctive feature is the USP structure responsible for deubiquitination [12]. The currently known substrates of USP10 include tumor suppressor protein p53 [13], cyclin CCND1 [14], sirtuin 6 [15], Yes‐associated protein1 [16], among others. Based on its specific deubiquitinating activity toward different substrates, USP10 is involved in controlling androgen receptor (AR), DNA damage response, energy metabolism, the development of vascular endothelial cells, stress granule formation, normal hematopoiesis, and various other cellular processes. This diversity of functions is also related to its dynamic subcellular localization. For instance, USP10 can translocate into the nucleus under stress conditions (such as DNA damage) to participate in various critical biological processes [3]. The current understanding of USP10 is partly derived from experiments conducted on genetically engineered mouse models. The knockout of USP10 leads to the death of mice due to bone marrow failure and pancytopenia [17]. This highlights its indispensable role in the body. Under physiological conditions, USP10 can directly deubiquitinate AR, functioning as a cofactor for androgen response [18]. In prostate cancer, which is characterized by elevated AR expression [19], overexpression of USP10 has been observed [20], which may indicate that the activity of USP10 has extended from physiological regulation to driving pathology. USP10 regulates protein homeostasis by directly affecting substrates or forming protein complexes. More specifically, USP10 deubiquitinates KLF4, which inhibits lung cancer cell growth [21]. The deubiquitination of Smad4, which is dependent on USP10, aggravates cardiac fibrosis after ischemia‐reperfusion injury [22]. Mounting evidence suggests that USP10 plays a vital role in neurological diseases, such as Alzheimer's disease (AD), Parkinson's disease (PD), and stroke. This underscores the importance of USP10 in the nervous system. However, no comprehensive summary of this research has been provided.

Multiple mechanisms regulate the activity of USP10. Studies have shown that USP13 can remove ubiquitin chains from USP10 through deubiquitination, enhancing its stability [23]. Additionally, phosphorylation of USP10 is necessary for regulating its subcellular trafficking [24]. In cardiomyocytes, forkhead box O4 (FOXO4) has been shown to negatively regulate the transcription of USP10, which induces cell apoptosis and oxidative stress [25]. Due to its important functions and multilevel regulatory mechanisms, USP10 has become a promising therapeutic target for the development of agents that affect USP10 activity, including Spautin‐1, Wu‐5, and DZNep.

Our paper reviews the structure and functions of USP10 and focuses on its regulatory role in neurological disorders. We also discuss the current development status of USP10 modulators and highlight the therapeutic potential of targeting USP10 in neurological diseases.

## 2 Literature Search Methods

**FIGURE 3:**
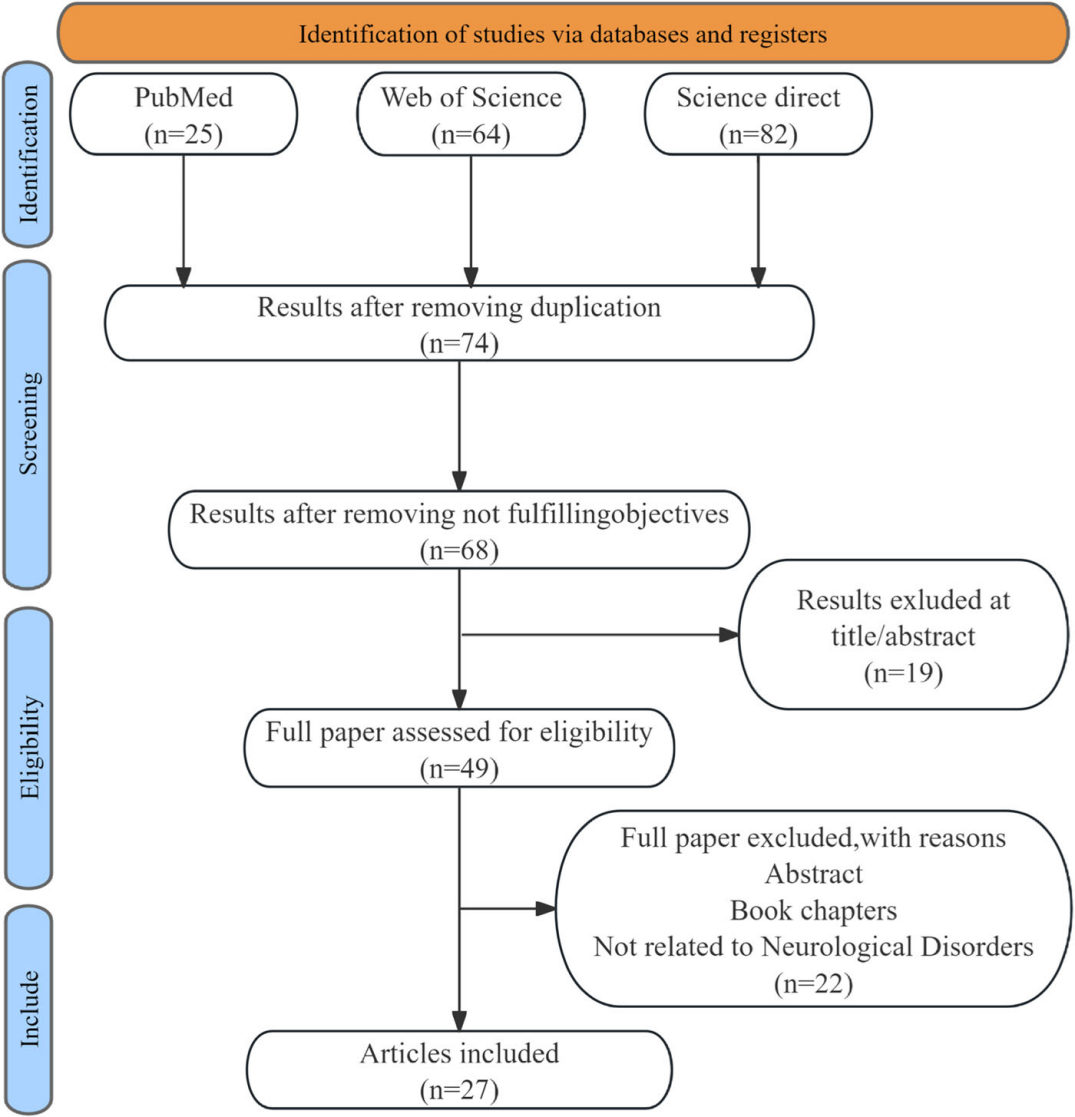
Search strategy. The flowchart illustrates the literature search and screening process for the study. An initial search was conducted across PubMed, Web of Science, and ScienceDirect. Following this, duplicate records and articles that did not meet the eligibility criteria were excluded. Ultimately, relevant studies were selected for further analysis.

To ensure comprehensiveness and reproducibility, the search strategy was strictly formulated in accordance with the guidelines of PRISMA [26]. We performed literature searches on PubMed, Web of Science, and Science Direct, with the medical subject headings (USP10, Nervous System Diseases). We limited our selected articles to English‐language studies from January 2015 to April 2025. Using the PubMed database as an example, we list our keywords for searching as follows: “(USP10 OR ubiquitin specific protease 10 OR USP10[Mesh]) AND (Nervous System Diseases OR Disease, Nervous System OR Nervous System Disease OR Nervous System Disorders OR Disorders, Nervous System OR Disorder, Neurologic OR Neurologic Disorder OR Neurological Disorders OR Disorders, Neurologica OR Neurological Disorder OR Nervous System Diseases[Mesh]).” Duplicate publications were removed, and the literature screening was conducted collaboratively by three researchers (Celemuge, J.Z., and Y.Y.); disagreements were resolved by discussion. Inclusion criteria included original research articles that evaluated the role of USP10 in neurological‐related pathologic processes in human or animal models. Book chapters, abstract‐only texts, articles in languages other than English, and irrelevant articles were excluded. The reference lists of all articles that were retrieved were further screened for additional eligible publications. Studies on the structure or organic properties of USP10 inhibitors were also included. Following a rigorous selection process, 27 articles were included in our study. The PRISMA flowchart illustrates the identification and screening process of articles related to the fundamental mechanisms of USP10 in neurological disorders (Figure 3).

## 3 Structure and Function of USP10

**FIGURE 4:**
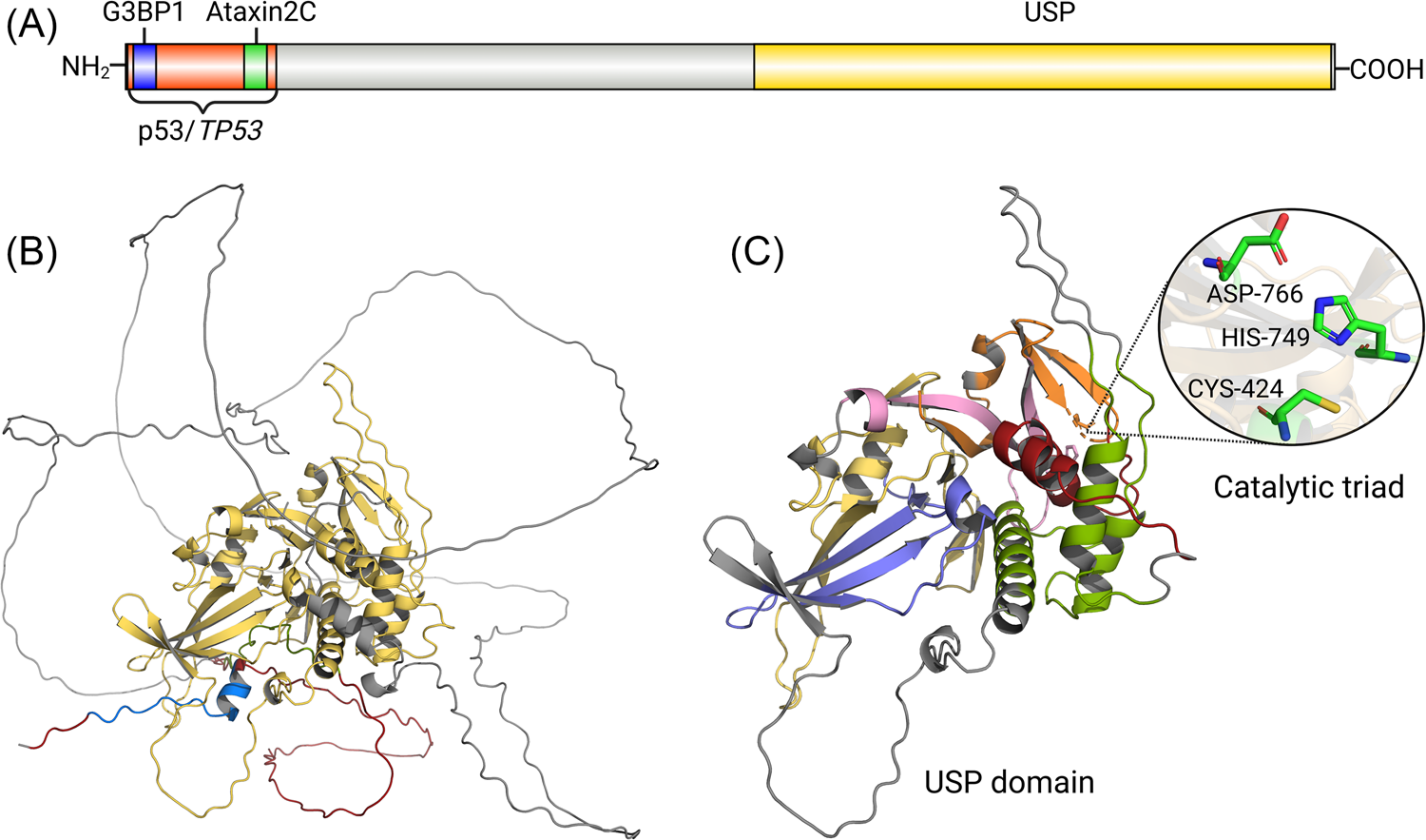
Structure of the USP10. (A) USP10, a 798‐amino acid protein, contains four structural domains: the USP catalytic domain (yellow), the Ataxin2C domain (green), the G3BP1‐binding domain (blue), and the p53‐binding domain (red). (B) USP10 full‐length model. (C) The six conserved boxes and insertions of USP10 are labeled and color‐coded: box 1 (red), box 2 (green), box 3 (blue), box 4 (yellow), box 5 (pink), box 6 (orange), and insertions (gray). The three key amino acids Cys424, His749, and Asp766 of the USP7 catalytic triad are highlighted in stick form B and C are from AlphaFold (alphafold.com) [32,33].

In eukaryotic cells, USP10 is highly conserved. The amino acid sequence of USP10 is approximately 99% similar between humans and mice [3]. The gene of USP10 is localized on chromosome 16q24.1 and encodes a protein containing 798 amino acids [27]. The relative molecular mass of USP10 is approximately 92.9 kDa [28]. The SOPMA online server was utilized to analyze the secondary structure of USP10 protein and found that alpha helices, extended strand structures, beta turn, and random coil conformation within USP10 account for 16.42% (131 AAs), 7.52% (60 AAs), 1.25% (10 AAs), and 74.81% (597AAs), respectively. Past studies have suggested that USP10 has two major domains, the USP domain and the Ataxin2C domain. With deeper analysis of USP10, researchers have identified sites where USP10 binds to p53 and G3BP1 [29−31] (Figure 4A). The AlphaFold‐predicted structure of USP10 (Figure 4B,C) shows the N‐terminal region as having random coils based on low pLDDT (predicted Local Distance Difference Test) scores, indicating insufficient information to predict precise structure [32,33].

The USP domain of USP10 comprises 381 residues (aa 415‐895) and can be divided into three subdomains: finger, thumb, and palm. The catalytic site is located at the interface between the thumb and palm subdomains and contains Cys‐His‐Asp/Asn residues, which form a catalytic triad. The finger subdomain is responsible for grasping distal ubiquitin in the ubiquitin chain during deubiquitination [34]. Ye et al. further divided the USP domain into six conserved boxes. In general, the catalytic Cys residues reside in box 1, catalytic His residues in box 5, and catalytic Asp/Asn residues in box 6. Box 3 and box 4 contain a zinc‐binding motif (Cys‐X‐X‐Cys), which facilitates folding of the USP core. It promotes interactions between motifs separated by hundreds of residues. The six conserved boxes provide five insertion sites, and different insertions can affect the function of USPs. Multiple sequence alignment (MSA) of all USP domains identified the six conserved boxes and insertions of USP10 (Figure 4C). The results of MSA suggest the catalytic triad of USP10 may consist of Cys424, His749, and Asp766 [35]. The function of the Ataxin2C domain has not been fully clarified, but it is currently believed to be linked to ataxia [12]. The discovery of these domains is critical for understanding the biological functions of USP10.

USP10 has distinct biological characteristics. First, USP10 has enzymatic activity. USP10 is a cysteine‐type endopeptidase that can hydrolyze the α‐peptide bonds within polypeptide chains by utilizing the sulfhydryl group of the cysteine residue at its active site. USP10 also exhibits thiol‐dependent isopeptidase activity, enabling it to remove ubiquitin from conjugated target proteins [23]. This is the most important feature of USP10.

Second, USP10 exhibits substrate dependency. The involvement of USP10 in various diseases suggests that its deubiquitination substrates are highly varied (Table 1). Nearly 70 proteins have been identified as substrates for USP10 deubiquitination. The role of USP10 in disease is directly tied to the function of its substrates rather than to USP10 itself. P53 is an important tumor suppressor that induces apoptosis and prevents abnormal cell proliferation [30]. Mutant p53 (mtp53) usually loses the tumor suppressor function of wild‐type p53 and may even promote new oncogenic functions [36]. USP10 acts on wild‐type p53 to inhibit the progression of multiple tumors, such as breast, lung, and thyroid cancers [37−39]. However, USP10 deubiquitinates mutant p53, which promotes the proliferation and metastasis of pancreatic cancer and p53‐mutant lung cancer [40, 41]. In addition, USP10 targets proliferating cell nuclear antigen and plays a carcinogenic role in hepatocellular carcinoma (HCC) [42]. As several USP10 substrates function as key signaling pathway factors, USP10 emerges as an upstream regulator of multiple signaling networks. USP10 deubiquitinates Notch1 intracellular domain (NICD1), and enhances its abundance and stability to positively regulate Notch signaling [43]. USP10 inhibits Smad4 degradation to promote TGF‐β signaling [44].

**TABLE 1:** The target proteins and downstream signaling pathways of USP10 in diverse diseases.

Disease	Substrates	Downstream signaling pathways
Lung cancer	EIF4G1, NSCLC, KLF4, PTEN, P53, P14ARF	AKT/mTOR
Colorectal cancer	XAB2, GRP78, P53, Axin1, ZEB1, NLRP7, MSI2, SIRT6	Wnt/β‐catenin, NF‐κB
Breast cancer	CD44, TCF4, Snail, ALK, IGF2BP1, P53	
Pancreatic carcinoma	FOXC1, DIRAS2, P53, PABPC1, N1ICD, PGK1, SOX21, YAP1	Wnt, MAPK, Notch
Prostate cancer	METTL13, PD‐L1, G3BP2, P53	P13K/AKT
Osteosarcoma	FASN, GSK3β, YAP1	
Multiple myeloma	SSRP1, CCND3	
Nasopharyngeal carcinoma	G3BP1, ATMIN	
Thyroid cancer	SIRT4, P53	
Ovarian cancer	G3BP1, P14ARF	
Esophageal cancer	PD‐L1, MOF, YAP, PARP1, ANLN	Wnt
Liver cancer	SNAI1, PCNA, YAP1, LKB1, Smad4, PTEN, AMPKα	TGF‐β, mTOR
Gastric cancer	TNFRSF10B, YBX1, DDX21, RFC2, P53	
Cutaneous melanoma	YTHDF2	
Myeloid leukemia	SKP2, P53, Rap1b, FLT3, SYK	Bcr‐Abl
Glioblastoma	RUNX1, CCND1	
Cardiac fibrosis	P53, Smad4	TGF‐β
Dilated cardiomyopathy	Smad4	TGF‐β
Myocardial infarction	NICD1	Notch
Cardiac hypertrophy	SIRT6	
Atherosclerosis	AMPKα, MKL1	AMPK
Chronic kidney disease	P53	
Acute kidney injury	FOXQ1	
Sepsis	GPX4, FOXQ1, NEMO	NF‐κB
Amyotrophic lateral sclerosis	P53	
Ischemic stroke	GRP78, YBX1, PGK1, NEMO	PINK1/Parkin, NF‐κB
Intracranial aneurysm	KLF4	
Alzheimer's disease	Tau	
Osteoporosis	FOXO1, NR3C1, SKP2	
Diabetic vascular calcification	AMPKα	
Endometriosis	Raf‐1	NEK/ERK

Finally, the distribution of USP10 is dynamic. USP10 is primarily localized in the cytoplasm [16], but it can translocate to the nucleus and other organelles with changes in cell state and environmental conditions. Under normal circumstances, USP10 deubiquitinates p53 in the cytoplasm and reverses p53 nuclear export and degradation. When DNA is damaged, USP10 moves into the nucleus and interacts with p53. The accumulation of USP10 in the nucleus prevents the cell from entering the cell cycle until the DNA damage is repaired [30]. These characteristics of USP10 enable it to coordinate stress responses across cellular compartments.

## 4 The Role of USP10 in Neurological Disorders

According to recent research, USP10 has been implicated in the pathogenesis of multiple neurological disorders, including AD, PD, spinal cord injury, glioblastoma (GBM), amyotrophic lateral sclerosis, and ischemic stroke (IS). Although these diseases differ in their pathological features and clinical manifestations, they are all associated with neuronal injury, neurodegeneration, or abnormal protein accumulation. USP10 plays a key role in regulating protein stability and influencing signaling pathways, and its dysfunction is an important factor in the pathogenesis of these neurological disorders.

### 4.1 USP10 in AD

The number of people worldwide with AD was estimated to be around 50 million as of 2020, and this figure is projected to rise to 152 million by 2050 [45]. Currently, it is widely accepted that senile plaques (SPs) formed by extracellular β‐amyloid (Aβ) aggregates, and neurofibrillary tangles (NFTs) formed by intracellular aggregates of hyperphosphorylated Tau proteins are the main pathological features of AD [46]. Notably, Aβ and Tau play a synergistic role in the pathological changes of AD [5, 47, 48]. Wei et al. found that USP10 expression was upregulated 1.6‐fold in the hippocampus of AD patients compared with normal‐aged controls [49]. This alteration in expression is detrimental to recovery from AD. The promotion of AD pathology by USP10 is manifested in two mechanisms, related to Aβ and Tau proteins.

#### 4.1.1 Positive Feedback Loop Between USP10 and Aβ

**FIGURE 5:**
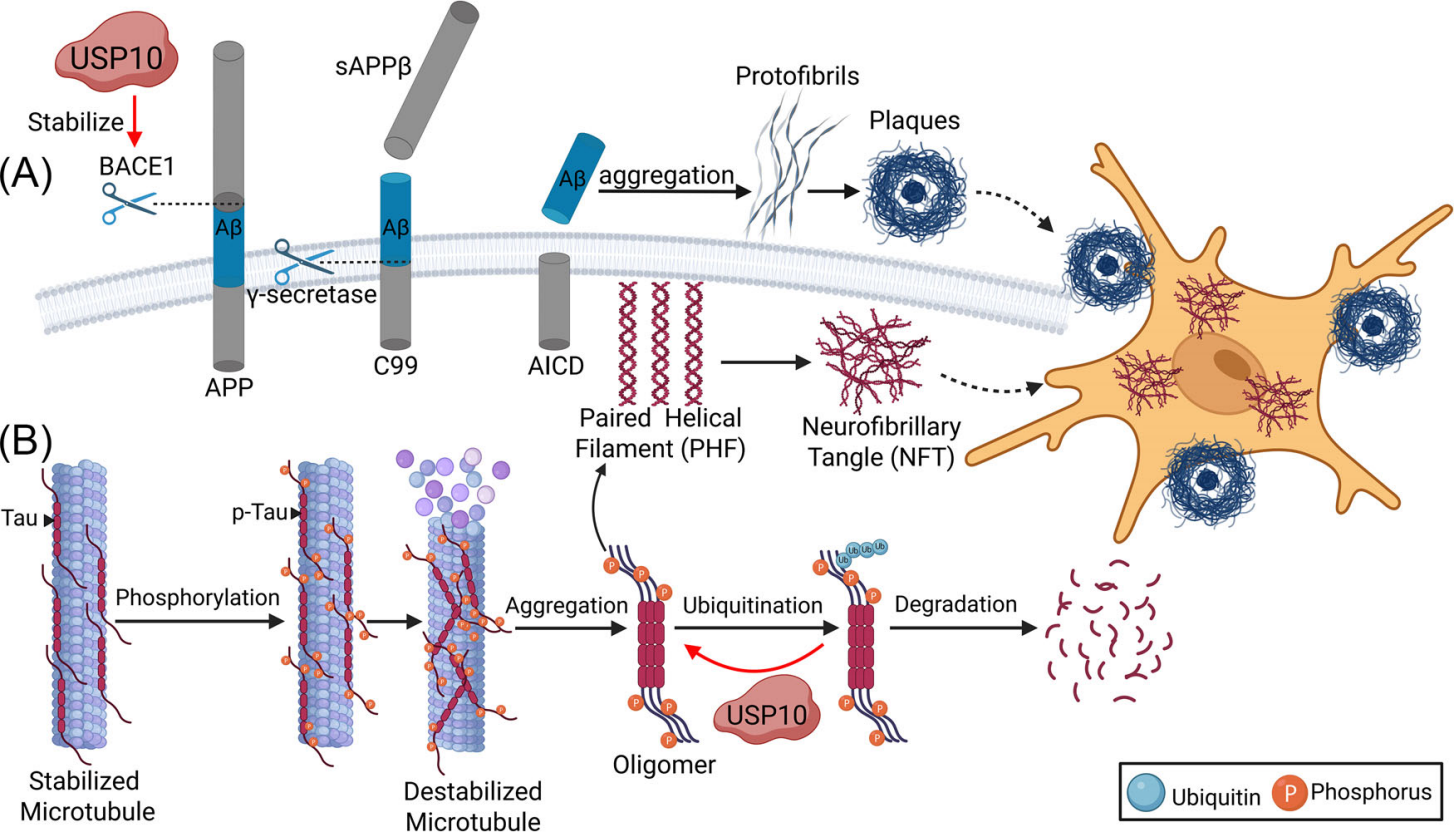
The mechanism of USP10 in the pathogenesis of Alzheimer's disease. (A) APP is cleaved by BACE1 and γ‐secretase to produce sAPPβ and C99, which eventually form AICD and Aβ. Aβ then aggregates to form protofibrils, and eventually transforms into plaques. USP10 may stabilize BACE1 and indirectly promote the formation of Aβ and plaques. (B) The abnormal phosphorylation of Tau protein produces p‐Tau, which loses its microtubule‐stabilizing function and subsequently aggregates into PHFs and NFTs. USP10 promotes this pathogenesis by deubiquitinating Tau, which increases Tau and p‐Tau levels. USP10 indirectly destabilizes microtubules, and facilitates NFT formation.

USP10 may be involved in the production of Aβ. Previous research has indicated that inhibiting USP10 activity leads to a reduction in Aβ secretion. The reduction of Aβ levels improves function related to learning and memory [50]. Consistent with these findings, Cai et al. also reported similar results. By analyzing magnetic resonance imaging (MRI) scan data and clinical data from AD patients and normal individuals, they identified five variant genes associated with hippocampal subregion volume. USP10 is one of these genes, and it is related to the granule cell and molecular layer of the dentate gyrus. The results of homology modeling, molecular docking, molecular dynamics (MD) simulations, and coimmunoprecipitation (Co‐IP) assays all reveal that USP10 interacts with β‐site amyloid precursor protein‐cleaving enzyme 1 (BACE1) [51]. BACE1, a β‐secretase enzyme, hydrolyzes amyloid precursor protein (APP) in the extramembrane region and generates sAPPβ and C99 (Figure 5A). The γ‐secretase enzyme cleaves C99 within its transmembrane domain, resulting in the formation of Aβ [52]. In the brains of AD patients, the expression and activity of BACE1 are elevated, which could promote Aβ production [53]. BACE1 is a direct substrate of USP8 and USP25, both of which enhance its stability. Being part of the same family, USP10 is likely to perform a comparable function, particularly in stabilizing BACE1 through deubiquitination, as observed with USP8 and USP25 [54, 55]. If this hypothesis is correct, USP10 regulates APP processing to promote Aβ generation. Aβ aggregates to form oligomers, which exhibit stronger neurotoxicity. These oligomers further aggregate to form amyloid fibrils, which progressively assemble into a dense core structure, ultimately resulting in the formation of SPs [56].

Elevated USP10 expression in AD is closely associated with Aβ. In N2a cells treated with Aβ42 oligomers, the levels of USP10 increased in a dose‐dependent manner [49]. These findings indicate that Aβ oligomers may act as the initiating factor to increase the expression of USP10 in the context of AD. Given that USP10 may enhance Aβ production, a vicious cycle is formed between USP10 and Aβ. This positive feedback mechanism may contribute to the pathological progression of AD. However, further research is necessary to validate this conclusion.

#### 4.1.2 USP10 is a Key Regulator of Tau Protein Stabilization and Aggregation

USP10 removes ubiquitin degradation signaling from Tau protein and enhances its stability (Figure 5B). This result elevates the overall level of Tau proteins (including soluble Tau and insoluble p‐Tau). The interaction between USP10 and Tau proteins is especially enhanced by toxic stimulation by Aβ42 oligomers [49]. P‐Tau has a diminished binding affinity to microtubule proteins, which can disrupt the cytoskeleton, interrupt axonal transport, and ultimately lead to neuronal death. Additionally, increased free p‐Tau levels lead to abnormal aggregation, fibril formation, and the formation of NFTs [57]. The findings presented above provide an alternative explanation for how Aβ facilitates the aggregation of Tau proteins and highlight the potential role of USP10 in the pathogenesis of AD [58].

Wei et al. engineered two peptides (Tau307‐326K and Tau341‐378K) that target the binding sites between USP10 and Tau. These peptides effectively weakened the USP10‐Tau interaction and reversed the abnormal accumulation of Tau and p‐Tau. Notably, the combined treatment with both peptides yielded greater effects compared to using either peptide alone [49]. This implies that interfering with the interaction of USP10 and Tau is a viable option for the future treatment of AD.

### 4.2 USP10 in PD

PD is the second most common neurodegenerative disease after AD [59]. It is primarily characterized by the degeneration of dopaminergic neurons and the development of intracellular aggregates known as Lewy bodies (LBs). These changes result in motor impairments such as resting tremors, rigidity, bradykinesia, and postural instability. Alpha‐synuclein (α‐syn) is the pathogenic protein in PD and the main component of LBs [60]. Takahashi et al. found that USP10 levels were increased in the amygdala of PD patients compared with controls [61]. Based on previous studies, USP10 may tend to exert a protective effect in the early stages of PD.

**FIGURE 6:**
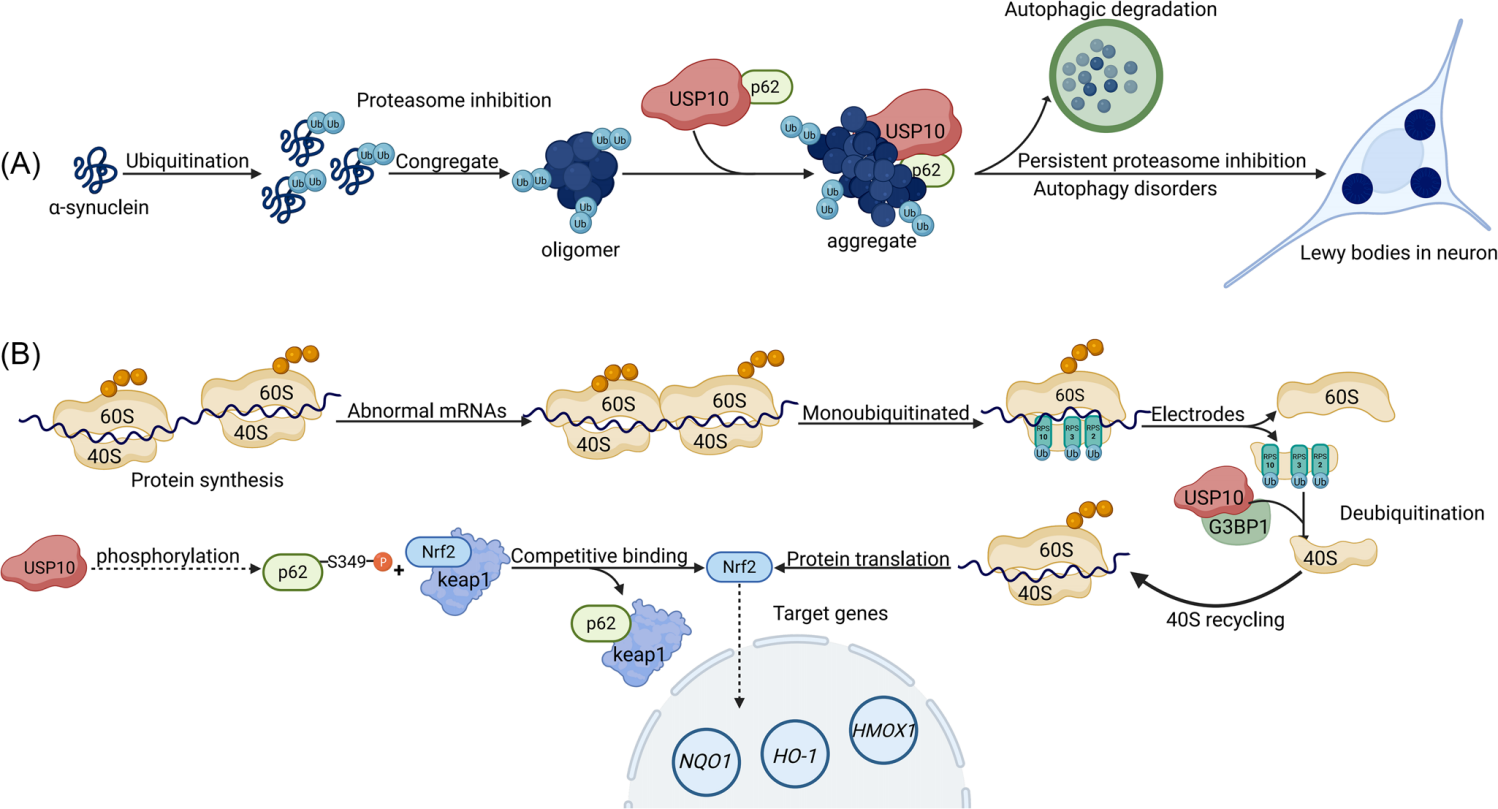
The role of USP10 in PD. (A) The ubiquitinated α‐syn causes neurotoxicity. P62 binds to the ubiquitin chains, forming p62 bodies, which is then degraded through autophagy. The direct binding of USP10 to p62 may facilitate this process and reduce the neurotoxicity of ubiquinated proteins. (B) Damaged mRNA causes ribosome collisions and eventually leading to the dissociation of the ribosome complex. USP10 enhances the stability of ribosomal proteins (RPS2, RPS3, and RPS10), which allows them to be recycled for new rounds of translation. This recycling process causes increased translation of Nrf2.

Excessive accumulation of ubiquitinated proteins is neurotoxic and induces neuronal apoptosis. This is an important pathological process in PD, which is often accompanied by proteasome system dysfunction. In general, aggregation of ubiquitinated proteins can diminish this neurotoxicity to some extent [62]. USP10 promotes the aggregation of ubiquitinated proteins. Consequently, USP10 alleviates the neurotoxicity of ubiquitinated proteins and inhibits cell apoptosis. This effect was independent of the deubiquitination activity of USP10. Specifically, the interaction between USP10 and p62 enhances p62‐dependent ubiquitinated protein aggregation (Figure 6A). The N‐ and C‐termini of USP10 are key regions for this interaction. Among the proteins that could induce aggregation by USP10 is α‐syn [61]. Most of the aggregates that form oligomers are cleared by the autophagy‐lysosome pathway [63]. In an emergency situation, p62 can bind to ubiquitin chains, leading to liquid−liquid phase separation, and subsequently form a gel‐like structure (p62 bodies). Around the p62 bodies, an isolation membrane is formed. p62 binds to LC3 or GABARAP on the isolation membrane, and the wetting effect between the p62 bodies and the isolation membrane leads to the selective isolation of p62 bodies and their client proteins into autophagosomes [64]. Therefore, larger protein aggregations, which form inclusion bodies, can be observed in cells, and LBs are an example of this [63]. This explains why USP10, p62, and α‐syn colocalize to LBs [61, 65]. This suggests that USP10 may exert a protective effect in PD by inhibiting the neurotoxicity of ubiquitinated proteins and promoting their clearance. Obviously, as the disease progresses, the protective effect is gradually weakened and overshadowed by the detrimental impact of excessive pathogenic accumulation. The targeted administration of USP10 remains of great value and can offer a distinctive advantage as an adjuvant therapy.

Owing to the abnormal loss of dopamine neurons, the treatment of PD often alleviates symptoms through the dopamine precursor levodopa. However, both experimental studies and clinical observations have demonstrated that long‐term use of levodopa has reduced therapeutic efficacy and a variety of complications, including dyskinesia and psychiatric symptoms [66]. This effect may be related to the production of reactive oxygen species by dopamine, which in turn promotes cell apoptosis [67]. In cells treated with dopamine, USP10 can counteract the adverse effects of dopamine by increasing the levels of the antioxidant factor nuclear factor erythroid 2‐related factor 2 (Nrf2) [68], which governs the expression of various antioxidant and cytoprotective genes [69]. By doing so, USP10 can combat oxidative stress, neuroinflammation, and mitochondrial dysfunction, thus potentially mitigating the pathological features associated with PD.

According to existing studies, USP10 may increase Nrf2 levels by promoting translation and inhibiting degradation (Figure 6B). First, USP10 promotes Nrf2 translation [68]. During translation, mRNA lesions trigger ribosomal decoding failure, leading to stalling and subsequent collision of trailing ribosomes. Ribosome‐associated quality control mechanisms employ ubiquitination to mark stalled complexes and allow their dissociation. The dissociated ribosome is degraded by the ubiquitin‐proteasome system. USP10 has been demonstrated to deubiquitinate ribosomal proteins RPS2, RPS3, and RPS10, and it functions as a complex with G3BP1 [70]. This activity inhibits ribosome degradation and ensures the recycling of ribosomal subunits to initiate a new round of protein translation.

Second, USP10 promotes the phosphorylation of p62 at S349 and activates the antioxidant activity of Nrf2 [68]. P62‐mediated Nrf2 activation depends on its phosphorylation at Ser349 (315 in mice) [71]. Phosphorylation of the Ser349 residue of p62 in the p62 bodies enhances the binding affinity of Keap1 to p62. This inhibits Keap1‐mediated degradation of Nrf2 in the cytoplasm and activates Nrf2. The retained Keap1, along with p62 bodies, is subsequently degraded by autophagy [64, 72]. The exact mechanism of how USP10 promotes p62‐Ser349 phosphorylation remains unclear. It is possible that USP10 is involved in the phosphorylation of p62 by regulating kinase activity. For example, Unc‐51‐like kinase 1 (ULK1) is a major kinase for Ser349 of p62 [71]. In osteosarcoma, USP10 promotes the expression of ULK1 mRNA and protein [73], which suggests that the pathway may operate through the USP10‐ULK1‐p62 axis. However, this conclusion requires further investigation in the context of PD. Notably, USP10 showed a tendency to negatively regulate Nrf2 in SH‐SY5Y cells without dopamine treatment, suggesting that the regulation of Nrf2 by USP10 may be dependent on different cell types and intracellular microenvironment [68].

In summary, USP10 participates in the pathological changes of PD by regulating processes such as autophagy, ribosome cycling, and oxidative stress in neurons. It can be reasonably hypothesized that USP10 may act as a nexus linking protein homeostasis and these cellular processes, collectively regulating PD pathogenesis. However, current studies on USP10 and PD have been conducted under inconsistent cellular conditions. Nevertheless, the significance of these findings in guiding future research remains undeniable.

### 4.3 USP10 in GBM

GBM is a malignant tumor originating from glial cells or precursor cells. It is the most common and most malignant primary brain tumor in adults, characterized by its high aggressiveness and rapid growth [74]. According to statistics, the survival rate is 35% in the first year after diagnosis, but it drops to 13.7% in the second year. Most patients do not survive more than 2.5 years, and only less than 5% of patients survive 5 years after diagnosis [75]. Existing studies suggest that USP10 is involved in the pathological progression of GBM as a promoter factor. In the cell function assay, overexpression of USP10 enhanced the proliferation and invasion ability of GBM cells, and the tumor volume also showed significant differences [76]. By analyzing tumor tissue samples from patients with GBM, researchers have found that USP10 expression can serve as a prognostic marker for GBM. The expression level of USP10 was inversely correlated with the survival rate of GBM patients [77]. Two proteins have been implicated in GBM degradation as direct substrates of USP10. These proteins are cyclin D1 (CCND1) and runt‐related transcription factor 1 (RUNX1) [14, 76]. Therapeutic strategies targeting USP10 and its substrates are expected to be novel therapeutic approaches for GBM.

#### 4.3.1 USP10/CCND1 May Promote the GBM Cell Cycle

**FIGURE 7:**
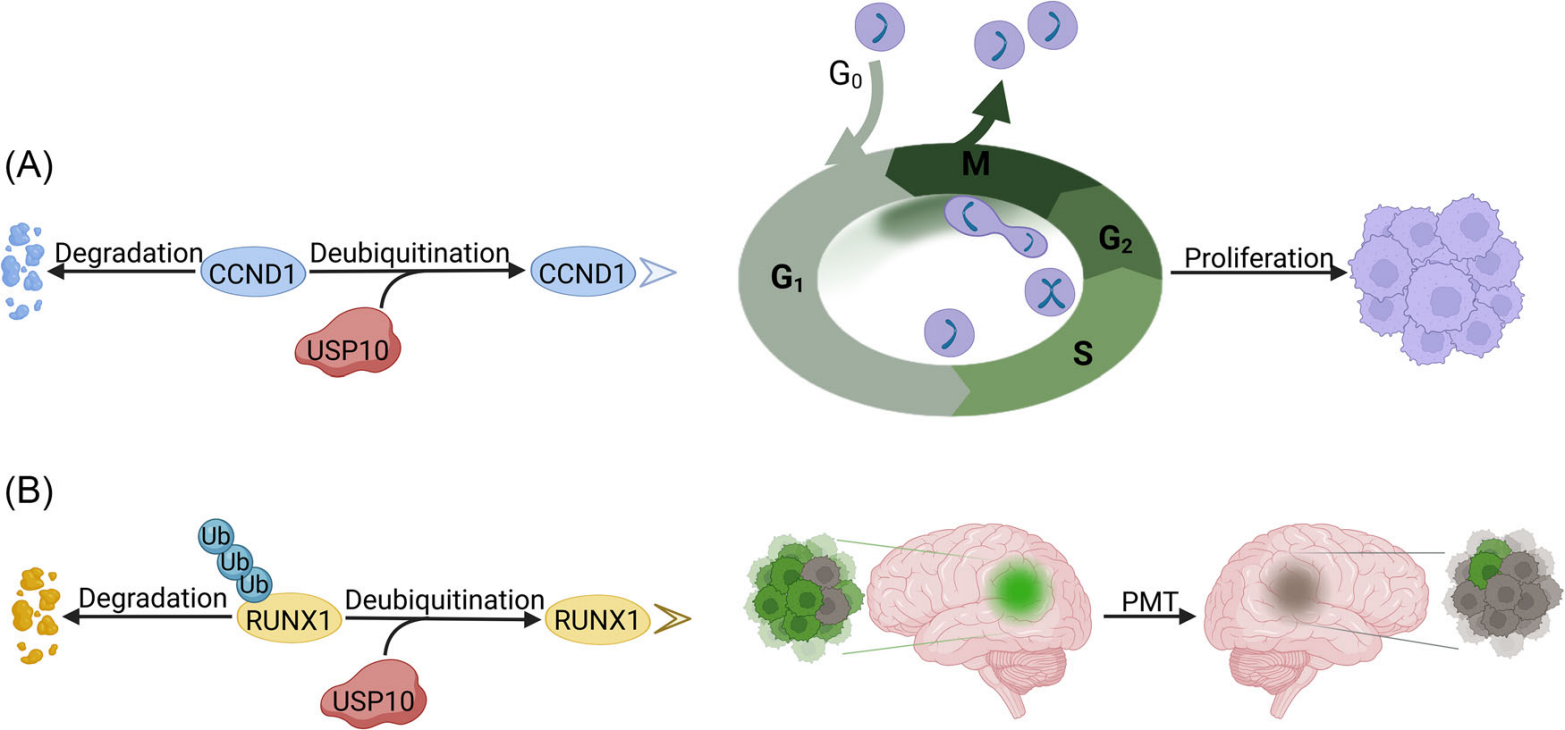
The influence of USP10 on the proliferation and proneural‐mesenchymal transition (PMT) of glioblastoma and PMT. (A) USP10 inhibits the degradation of CCND1 by deubiquitination. Furthermore, CCND1 facilitates the transition of glioblastoma cells from the G1 to the S phase of the cell cycle, which in turn enhances cell proliferation. (B) USP10 removes the ubiquitin linkage at Lys48 of RUNX1, and enhances the stability of RUNX1. RUNX1 promotes the transformation of GBM from the PN subtype to the MES subtype. Cells with PV features are shown in green, while those with MES features are depicted in gray.

CCND1 is a key factor that controls the cell cycle. It can help to trigger the cell cycle transition from the G1 to S phase [78]. Previous studies have demonstrated that the expression level of CCND1 shows an upward trend with the deterioration of glioma. The upregulation of CCND1 can predict poor prognosis in GBM patients [79]. USP10 deubiquitinates CCND1 and inhibits its K48‐linked polyubiquitination (Figure 7A). Therefore, it extends the half‐life of CCND1 from 1 to 4 h. Sun et al. found that the USP10/CCND1 axis promotes cell proliferation in GBM by advancing the cell cycle. Knockdown of USP10 induces cell apoptosis by arresting the cell cycle in the G1 phase. Acevaltrate has the same effect by inhibiting the USP10/CCND1 axis [14].

#### 4.3.2 USP10/RUNX1 Is Implicated in Malignant Transformation of GBM

Based on gene expression profiles and molecular features, GBM is classified into three subtypes, namely, proneural (PN), classical (CL), and mesenchymal (MES). Among them, the MES subtype is the most aggressive and malignant form [80]. The shift from PN to MES subtype, called the proneural‐mesenchymal transition (PMT), causes GBM to become more invasive and drug‐resistant [81, 82]. USP10 expression was highest in the MES subtype. USP10 is an upstream regulator of RUNX1 (Figure 7B). The N‐terminal 1–206 fragment of USP10 interacts with the N‐terminal 1–204 amino acid sequence of RUNX1. USP10 stabilizes RUNX1 by removing ubiquitin linked to Lys48. The USP10/RUNX1 axis facilitates the occurrence of PMT and advances the malignant progression of GBM [76]. However, the specific downstream pathways need to be further explored.

### 4.4 USP10 in IS

Stroke is the second leading cause of death in the world, causing about 7 million deaths each year. Moreover, stroke survivors also confront high rates of disability. IS is the most common type of stroke, accounting for approximately 65% of all stroke cases [83]. Age is a significant risk factor for IS. The risk approximately doubles with each 10‐year increase in age. With the increasing trend of global population aging, the challenge of preventing and treating IS will continue to increase [84]. IS is mainly caused by cerebral vascular occlusion and leads to ischemia and hypoxia in brain tissue. It then causes neurological dysfunction, which manifests as motor, sensory, language, cognitive, and other abnormalities [85]. Although restoring blood flow is necessary to treat IS, reperfusion may further aggravate damaged neurovascular and brain tissue [86]. In these processes, many stress responses are triggered, including the inflammatory response, mitochondrial autophagy, endoplasmic reticulum stress (ERS), and apoptosis, among others [87]. It has been demonstrated that USP10 expression decreases in a time‐dependent manner in a mouse model of IS. USP10 expression reached its lowest point at 72 h and then gradually increased. The deficiency of USP10 accelerates cerebral ischemic injury [88, 89]. Further studies suggest a protective role of USP10 in the progression of IS brain injury. This suggests that the upregulation of USP10 is a potential measure for the treatment of cerebral ischemic injury in the future.

#### 4.4.1 USP10 and Inflammatory Response

**FIGURE 8:**
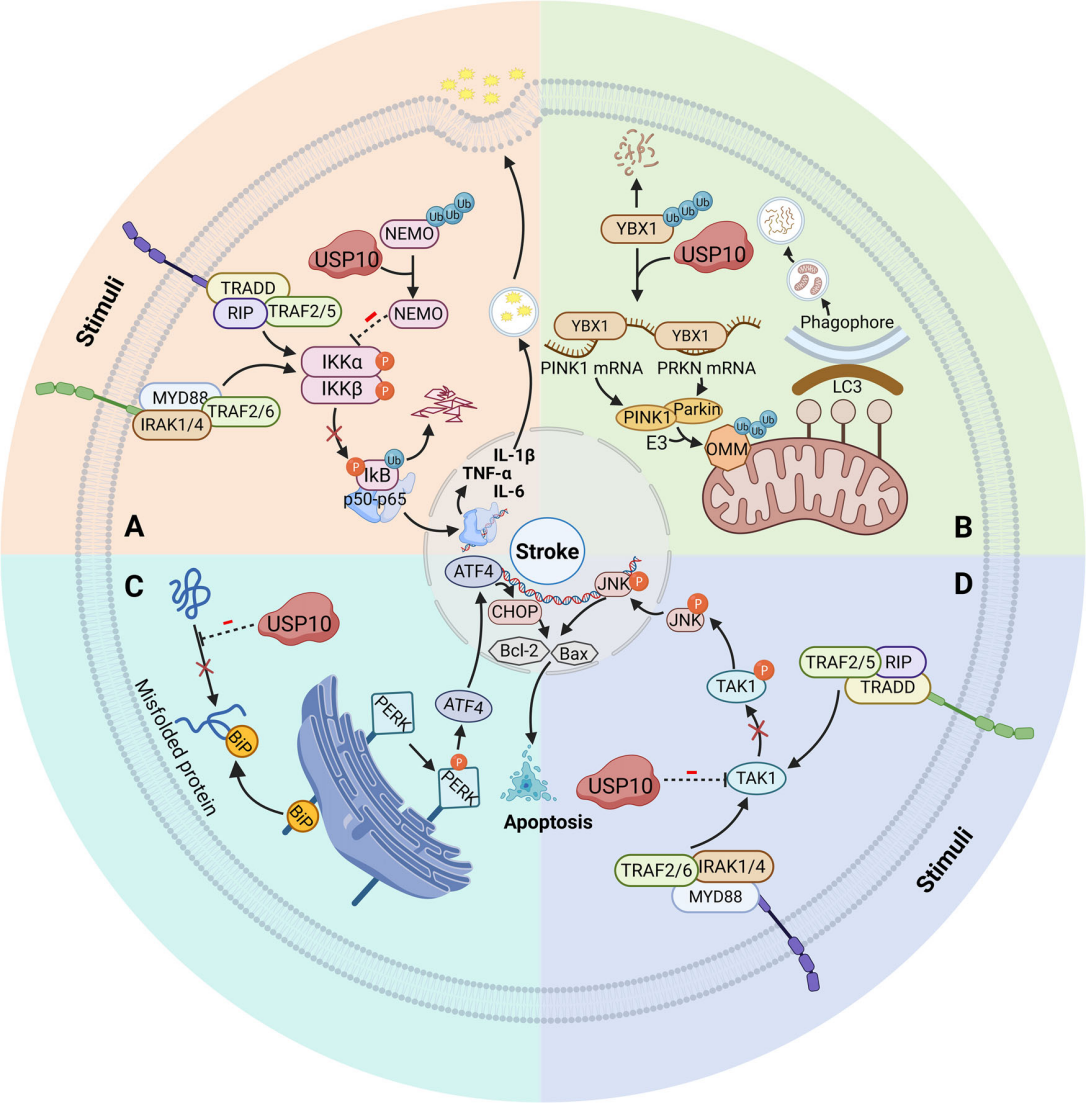
USP10 in ischemic stroke. (A) USP10 deubiquitinates NEMO. NEMO is unable to form a complex with IKKα and IKKβ, which inhibits the phosphorylation of downstream IκB. IκB is degraded by the proteasome. P50/p65 dimer cannot be transported into the nucleus, leading to reduction in the production of inflammatory factors. (B) USP10 enhances YBX1 stability, enabling its binding to and stabilization of PINK1/PRKN mRNAs to facilitate PINK1/Parkin‐dependent mitophagy. (C) USP10 inhibits the expression of Bip protein. PERK cannot be separated from Bip, which prevents its activation. This process reduces ERS. It is manifested as inhibiting cell apoptosis. (D) USP10 suppresses TAK1 phosphorylation and inhibits JNK pathway‐mediated apoptosis. The specific manifestation is an increase in Bcl‐2 and a decrease in Bax.

The nuclear factor kappa B (NF‐κB) pathway plays an important role in mediating inflammatory responses. In the canonical NF‐κB pathway, stimulating factors activate cell surface receptors (Figure 8A). Upon reception of the signal, IκB (inhibitor of NF‐κB subunit beta) kinase (IKK) phosphorylates IκB at specific serine sites. This leads to polyubiquitination and proteasomal degradation of IκB. Subsequently, NF‐κB dimers (usually p50/p65) are released and activated. The translocation of p65 and p50 into the nucleus activates κB‐dependent genes, leading to the production of inflammatory factors that contribute to the progression of IS injury [87, 90]. This process can be suppressed by USP10. USP10 removes the nondegrading ubiquitin signal of NEMO by deubiquitination [88]. NEMO, along with IKKα and IKKβ, forms the IKK complex. Its ubiquitination is essential for IKK complex assembly and activation [91]. USP10 inhibits the NF‐κB pathway by blocking NEMO‐mediated IKK activation. As a result, the levels of proinflammatory cytokines such as TNF‐α, IL‐1β, and IL‐6 are reduced. Additionally, USP10 can also inhibit the activation of astrocytes, which further reduces the release of inflammatory factors [88]. These mechanisms are crucial for maintaining the stability of the central nervous system and promoting recovery after ischemic injury.

Another study showed a different explanation for how USP10 inhibits the NF‐κB pathway [89]. TGF‐beta‐activated kinase 1 (TAK1) acts as an upstream kinase that activates the IKK complex [92]. USP10 interacts with TAK1 and inhibits TAK1 phosphorylation. In consequence, the NF‐κB pathway is attenuated, leading to reduced release of proinflammatory factors [89]. However, the specific regulatory mechanisms need to be further explored.

#### 4.4.2 USP10 and Mitochondrial Autophagy

Lin et al. and Li et al. found that USP10 inhibited ubiquitination‐dependent degradation of Y‐box binding protein 1 (YBX1) in gastric cancer models and in oxygen‐glucose deprivation/reoxygenation (OGD/R) in vitro models, respectively [93, 94] (Figure 8B). YBX1 is a highly conserved cold shock protein. It exhibits extensive nucleic acid binding capabilities and can bind to DNA and RNA [95]. YBX1 directly binds to *Pink1* and *Prkn* (encoding the E3 ubiquitin protein ligase Parkin) and enhances their stability [96]. The expression of PTEN‐induced putative kinase 1 (PINK1) and Parkin is enhanced. In the OGD/R model [94], USP10 overexpression upregulates LC3II/I (an autophagy marker) and mitochondrial PINK1 and Parkin; knockdown of YBX1 reversed the effect caused by USP10 overexpression. In mammals, the PINK1/Parkin pathway is the most canonical route of mitochondrial autophagy, vital for removing damaged mitochondria and maintaining mitochondrial homeostasis. Activation of this pathway provides neuroprotection against cerebral ischemia‐reperfusion injury (CIRI) [97, 98]. PINK1/Parkin ubiquitinates outer mitochondrial membrane (OMM) proteins. Autophagy receptor proteins recognize OMM and recruit LC3 to localize damaged mitochondria to the phagophore [99]. In summary, the USP10/YBX1 axis plays a neuroprotective role in CIRI by regulating the PINK1/Parkin pathway and activating mitochondrial autophagy.

#### 4.4.3 USP10 and Apoptosis

More and more studies have found a significant role of USP10 in inhibiting apoptosis. For example, USP10 can alleviate acute myocardial infarction injury by inhibiting myocardial cell apoptosis [25]. USP10 suppresses apoptosis and enhances multiple myeloma cell proliferation by deubiquitinating cyclin D3 (CCND3) [100]. In CIRI, the antiapoptotic effect of USP10 was manifested as an increase in the level of antiapoptotic protein Bcl‐2 (B cell leukemia/lymphoma 2) and a decrease in the levels of apoptotic proteins Bax (BCL2‐associated X protein) and cleaved‐caspase‐3 [89, 101]. USP10 can achieve this result in two ways.

First, USP10 alleviates neuronal apoptosis by downregulating ERS [101] (Figure 8C). CIRI triggers abnormal accumulation of unfolded or misfolded proteins, which ultimately leads to ERS. Mild ERS can help restore cell homeostasis, but excessive or long‐term ERS can aggravate neurological deficits in patients [102]. Glucose‐regulated protein 78 (GRP78) and C/EBP homologous protein (CHOP) are the classical markers and key proteins of ERS‐mediated apoptosis. GRP78, also called Bip, is an endoplasmic reticulum molecular chaperone protein. During ERS, GRP78 dissociates from the protein kinase RNA‐like endoplasmic reticulum kinase (PERK). PERK is activated and upregulates CHOP to induce apoptosis. CHOP upregulates the expression of proapoptotic proteins while downregulating the expression of antiapoptotic proteins [102, 103]. USP10 decreases the expression of GRP78 and CHOP and inhibits ERS‐induced apoptosis [101]. However, the specific mechanisms of USP10 to regulate ERS have not been clearly defined. A similar effect of USP10 on ERS has also been found in pancreatic cancer. Bhattacharya et al. also found that USP10 deubiquitinates RPS2 and RPS3 in this process [104]. The possibility that USP10 inhibits ERS by promoting ribosome recycling and optimizing protein folding cannot be excluded.

Second, TAK1 is a downstream target of USP10, as previously described. USP10 inhibits TAK1 phosphorylation and affects downstream pathways regulated by TAK1, including the JNK signaling pathway [89, 105] (Figure 8D). TAK1/JNK‐mediated apoptosis is widespread in various organs and tissues with ischemia‐reperfusion injury, such as the liver, myocardium, and kidney. TAK1 activates downstream JNK, which then translocates to the nucleus and influences the transcription of apoptosis‐related genes [105−107]. By inhibiting these processes, USP10 plays a crucial role in protecting neural cells from CIRI.

## 5 Exploration and Application of USP10 Targeted Modulators

Given the different roles of USP10 in various diseases, the targeted therapy of USP10 shows promising prospects. However, due to the high activity of DUBs and the overlapping of substrates, there are still many challenges in translating research results at the cellular level and in animal models into effective clinical treatments. To date, only a few USP10 modulators have been identified (Table 2). In addition, there are also several modulators that indirectly influence USP10 activity.

**TABLE 2:** Details of USP10 modulators.

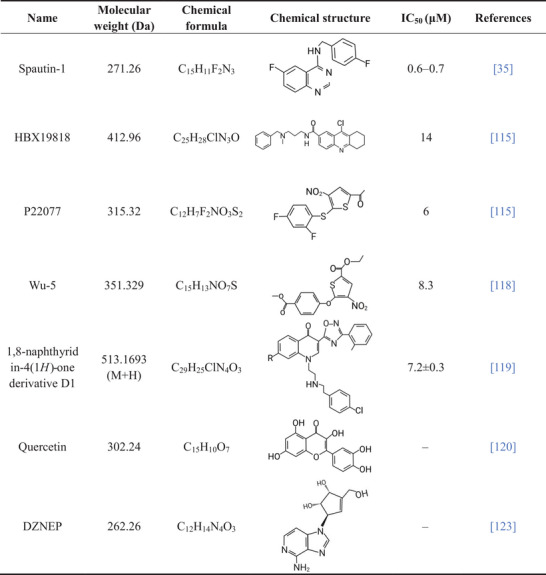

The small molecule inhibitor Spautin‐1 was initially identified as an autophagy inhibitor. It can target USP10 and USP13 with a half‐maximal inhibitory concentration (IC50) of 0.6−0.7 µM [23]. Spautin‐1 inhibits the deubiquitination ability of USP10, and subsequently attenuates the proliferation and invasion of many cancers, including osteosarcoma, hepatocellular carcinoma, and prostate cancer [42, 73, 108]. Spautin‐1 also plays a key role in the treatment of neurological diseases. Downregulation of USP10 by Spautin‐1 inhibits GBM proliferation and malignant transformation [76, 109]. Treatment with Spautin‐1 significantly extends the lifespan and enhances associative learning capability in *Caenorhabditis elegans* models of AD [110]. Spautin‐1 also alleviates cognitive deficits, memory impairment, and anxiety‐like behaviors in mice subjected to brain injury [111, 112]. However, further comprehensive studies on Spautin‐1 are needed to obtain sufficient evidence for clinical trials.

Recent studies have found that HBX19818 and P22077 can also inhibit the DUB activity of USP10, with IC50 values of 14 and 6 µM, respectively. This effect has potential therapeutic implications for acute myeloid leukemia (AML) [113]. However, these two compounds also showed inhibitory effects on USP7 [114, 115]. Wu‐5 is a novel USP10 inhibitor with an IC50 of 8.3 µM. Wu‐5/USP10 also has therapeutic value in AML [116]. Lu et al. used protein structure prediction, molecular dynamics simulations, and virtual screening to reveal that the 1,8‐naphthyridin‐4(1H) derivative D1 is a USP10‐targeting inhibitor. Compared with other inhibitors, D1 showed significant selectivity. By inhibiting the deubiquitination function of USP10, D1 promotes the ubiquitination and degradation of its substrate, YAP protein, and then downregulates p53 and its downstream protein, p21 [117]. The inhibitory mechanism of USP10 by quercetin, a natural flavonoid, and CNYP, a traditional Chinese medicine, remains unclear. Existing research has highlighted their potential roles in promoting apoptosis and modulating inflammatory responses [118, 119]. UbV.10.1 is a protein or polypeptide complex that has a high affinity for USP10 and inhibits its activity. It was identified by screening a phage display ubiquitin variant (UbV) library. The overexpression of UbV.10.1 promotes p53 translocation from the nucleus to the cytoplasm. Subsequently, it leads to p53 degradation [120]. The therapeutic value of the above inhibitors in neurological diseases has not been explored.

The S‐adenosylhomocysteine hydrolase inhibitor 3‐deazaneplanocin A (DZNep) stabilizes the p53 protein by upregulating the expression of USP10, which can activate p53 signaling pathways. P53 induces cell cycle G1 phase arrest and apoptosis. DZNep has potential value in the treatment of thyroid tumors [121]. Vagus nerve stimulation (VNS) plays a neuroprotective and anti‐inflammatory role in IS by regulating USP10. The study found that VNS intervention at 30 min, 24 h, and 48 h after stroke significantly upregulated the expression of USP10 in the brain. Subsequently, VNS reduced cerebral infarct volume and improved neurological deficits [88].

Although research on USP10 modulators has made significant progress, the field is still in its early stages and faces numerous challenges in terms of therapeutic potential and clinical translation. One of the key issues is the insufficient selectivity of the inhibitors. Most of the reported inhibitors, such as Spautin‐1 and P22077, target not only USP10 but also simultaneously act on other DUBs, including USP13 and USP7 [23, 122]. This multitarget characteristic may lead to off‐target effects and unpredictable toxicity, severely limiting their clinical application prospects. Additionally, the majority of inhibitors have only been tested in vitro, and their in vivo efficacy and detailed pharmacokinetic properties are still unclear. Especially, if one wants to develop inhibitors that can effectively cross the blood−brain barrier, it requires systematic considerations regarding solvent selection, formulation development, and physicochemical property optimization based on structure. P22077 has undergone in vivo experiments, but it requires frequent high‐dose administration to maintain efficacy [113], which not only reflects the problem of its short in vivo half‐life, but also indicates potential safety risks and resistance hazards in long‐term use. The proteolysis‐targeting chimeras (PROTACs) technology utilizes the intracellular ubiquitin‐proteasome system to selectively degrade disease‐related proteins and has become a new focus in drug development. Compared with traditional inhibitors, PROTACs have advantages such as overcoming drug resistance, high selectivity, and long‐lasting efficacy [123]. Although this technology has not yet been applied to USP10 and its family proteins, it points the way for future research. Developing specific PROTACs that specifically target USP10 and induce the degradation of USP10 to treat diseases associated with its abnormal expression is undoubtedly a promising new strategy.

## 6 Conclusion and Perspectives

As a DUB, USP10 affects many cellular processes by reversing ubiquitination, such as cell proliferation, inflammatory response, and apoptosis. Based on the abundance of substrate proteins, USP10 plays an important role in physiology and disease. Accumulating evidence highlights the therapeutic potential of USP10 in neurological disorders. The multifaceted roles of USP10 in neurological disorders present both intriguing therapeutic opportunities and significant research challenges. Perhaps the most striking controversy is its opposing functions in different diseases: USP10 may act as a pathogenic driver in AD and GBM by promoting the stability of proteins like Tau and BACE1 or CCND1 and RUNX1. In stark contrast, it may play a protective role in PD and IS by facilitating the clearance of toxic protein aggregates, enhancing antioxidant responses via Nrf2, and suppressing inflammatory and apoptotic pathways. This dichotomy underscores that the biological outcome of USP10 activity is profoundly context‐dependent, determined by cell type, subcellular localization, disease stage, and most importantly, the specific substrates it engages within a given pathological microenvironment.

Several critical knowledge gaps must be addressed to reconcile these opposing roles and translate findings into targeted therapies. First, the cell‐type‐specific functions of USP10 within the complex environment of the nervous system remain largely unexplored. It is unknown whether USP10 operates similarly in neurons, astrocytes, and microglia, or if its deubiquitinating activity has distinct consequences in each cell type that contribute differentially to disease pathogenesis. It is necessary to explore the expression pattern and function of USP10 in different cell types, across different developmental processes, pathological stages, and brain regions. Second, the precise upstream regulatory mechanism by which USP10 exerts its effects in various neurological disorders remains unclear. Identifying these is crucial for a deeper understanding of the impact of USP10 on diseases and its fundamental physiological functions. The regulatory effects of USP10 on the nervous system make it an attractive therapeutic target for disease intervention. Several small‐molecule inhibitors targeting USP10 have been discovered. Some of them have shown therapeutic potential in neurodegenerative diseases, but there are still many limitations that need to be further explored. This limitation is partly due to the lack of a clear USP10 structure. It is a crucial foundation for identifying new substrates, uncovering novel physiological roles, and designing targeted modulators. Elucidating the full‐length structure, especially the dynamic interplay between its N‐terminal domain and the catalytic USP domain, should be a priority. The highly conserved catalytic domains of the USP family allow them to be simultaneously targeted by covalent inhibitors, resulting in cross‐reactivity. The issue of multiple target inhibition remains the highest priority. Selecting allosteric inhibitors or complex‐specific inhibitors may be a more favorable strategy. Future efforts should also focus on developing PROTACs specifically for USP10, or engineering UbVs with higher specificity, to achieve targeted degradation or inhibition without affecting other DUBs. Several natural active ingredients and traditional formulations have shown the potential to modulate USP10. This provides more possibilities for future research directions.

In this review, we have identified USP10 as a key participant in a variety of neurological disease processes. The in‐depth study of its biological function and targeted regulation is extremely valuable.

## Author Contributions

**Celemuge**: writing – original draft, writing —review and editing. **Hongying Sun**: Funding acquisition, project administration, writing – review and editing. **Jia Zhang**: writing – review and editing, resources. **Yang Yang**: writing – review and editing, investigation. **Jian Mao**: writing – review and editing. **Cheliger**: writing – review and editing.

## Funding

This work was supported by the Science and Technology Program of the Joint Fund of Scientific Research for the Public Hospitals of Inner Mongolia Academy of Medical Sciences (No. 2024GLLH0536).

## Conflicts of Interest

The authors declare that they have no known competing financial interests or personal relationships that could have appeared to influence the work reported in this paper. Authors declare that they did not use any Artificial Intelligence Generated Content (AIGC) tools to generate content.
